# Effects of balance training in addition to auxiliary activity on balance function of patients with stroke at high risk for falls

**DOI:** 10.3389/fneur.2022.937305

**Published:** 2023-01-05

**Authors:** Huiqun Tan, Zhaohui Gong, Sisi Xing, Lanhua Cao, Huan Liu, Lijun Xu

**Affiliations:** ^1^Department of General Practice, Huangshi Central Hospital, Affiliated Hospital of Hubei Polytechnic University, Edong Healthcare Group, Huangshi, China; ^2^Hubei Key Laboratory of Kidney Disease Pathogenesis and Intervention, Huangshi, China; ^3^Department of Geriatrics, Huangshi Central Hospital, Affiliated Hospital of Hubei Polytechnic University, Edong Healthcare Group, Huangshi, China; ^4^Department of Neurology, Huangshi Central Hospital, Affiliated Hospital of Hubei Polytechnic University, Edong Healthcare Group, Huangshi, China; ^5^Department of Spine Surgery, Huangshi Central Hospital, Affiliated Hospital of Hubei Polytechnic University, Edong Healthcare Group, Huangshi, China; ^6^Department of Neurosurgery, Huangshi Central Hospital, Affiliated Hospital of Hubei Polytechnic University, Edong Healthcare Group, Huangshi, China; ^7^Department of Radiology, Huangshi Central Hospital, Affiliated Hospital of Hubei Polytechnic University, Edong Healthcare Group, Huangshi, China

**Keywords:** balance training, activity auxiliary, stroke, balance function, stroke fall

## Abstract

**Objective:**

The aim of this study was to investigate the effect of balance training in addition to auxiliary activity on the balance function of patients with stroke at high risk for falls.

**Methods:**

A total of 112 patients with stroke at high risk for falls in our hospital from inception to January 2020 to December 2020 were selected as the research objects who were equally divided into the control group and study group according to the random number table method. Patients in the control group were intervened with auxiliary activity, and the patients in the study group received additional balance training for auxiliary activity. The balance function, lower extremity motor function, fall risk, walking ability, and other indicators were compared between the two groups of patients before and after treatment. The hospitalization time and the frequency of falls 3 months after discharge were also compared between the two groups.

**Results:**

Before treatment, there was no significant difference in balance function, lower extremity motor function, fall risk, and walking ability scores between the two groups (*P* > 0.05). After 3 months of treatment, the balance function, lower extremity motor function, and walking ability scores of the study group were significantly higher than those of the control group, with the fall risk score significantly lower than that of the control group (*P* < 0.05). Evidently, lower hospitalization time and the frequency of falls in the 3-month follow-up of the study group were observed than those in the control group (*P* < 0.05). However, there was no significant difference in the frequency of falls between the two groups during hospitalization (*P* > 0.05).

**Conclusion:**

Our results indeed revealed that balance training in addition to auxiliary activity elicited beneficial outcomes in terms of effectively improving the balance function and walking ability of patients with stroke at high risk of falling, which may have the potential for wide clinical application.

## Introduction

Cerebral stroke is defined as a cerebrovascular accident consisting of a life-threatening condition of the clinical nervous system with high morbidity, disability, and mortality, seriously affecting the health of patients (1). According to the latest Global Burden of Disease Study, the overall risk of stroke in our country is 39.9%, with a 75% disability rate and a 6.8% recurrence rate within 3 months, which has become the second leading cause of death worldwide (1, 2). Patients with stroke lose the control function of the lower center due to high central lesions, which leads to sensory or motor impairment. The body's center of gravity shifts to the healthy side, and the weight-bearing ability and stability of the affected limb are decreased to varying degrees (3). The patients cannot control the body's center of gravity and maintain normal posture, thus, affecting their balance function. Relevant data reported that patients with stroke had significantly lower stability when walking than normal people due to their inability to control body excursion and peak trunk velocity. It has a high incidence of falls, ranging from 23 to 50% within 6 months after stroke (4, 5). As a common complication of patients with stroke, falls can cause soft tissue trauma, head injury, and fracture, which leads to limitations in mobility and a reduced ability to independently perform daily activities. Patients with stroke have more than twice the risk of falls and secondary injuries compared with their peers without stroke (5). Repeated falls will bring different degrees of physical and psychological damage to patients, and in severe cases, fractures, bedridden, limb paralysis, craniocerebral injury, and even death may occur due to serious complications (5). Therefore, patients with stroke at high risk of falling should seek early and appropriate intervention to prevent falls and a series of complications due to falling, thus promoting their rehabilitation.

The main cause of falls in patients with stroke is balance dysfunction. It typically affects their control of their gravity center and their ability to restore their balance to avoid falling. The ability to balance is the basis for patients to perform all functional activities, including sitting, standing, and moving, by improving which patients with stroke can effectively prevent the occurrence of falls (6). Scientific and prompt management to avoid falling is thereby imperative for clinical practice.

Balance training can improve the balance ability of patients with stroke and prevent or reduce the occurrence of falls (6). The auxiliary activity is an activity with an auxiliary device that can assist patients to get out of bed and prevent falls in patients with stroke (7). The effect of rehabilitation using balance training or auxiliary activity solely has been reported in earlier studies, whereas limited data exist regarding the application of balance training in addition to auxiliary activity in patients with stroke at high risk for falls (8). Moreover, there are no abovementioned reports in China. In this study, we aimed to explore the effect of balance training in addition to auxiliary activity on the balance function of patients with stroke at high risk for falls.

## Methods

### Participants

The current prospective randomized controlled study included patients with stroke at high risk for falls in our hospital from January 2020. The patients enrolled were equally allocated into two groups according to the random number table method. Patients in the control group received auxiliary activity interventions, and patients in the study group received additional balance training for auxiliary activity. The sample size was calculated according to a previous study (9). *n* = (μ1-α/2+μ1-β) 2S2 (1+1/k)/(μt-μ) 2, thus, it was determined that the sample size of this study was n = 75×1/ (1–0.15) = 56.12 ≈ 56. Therefore, a final total of 112 patients with post-stroke at high risk of falling admitted to our hospital from January 2020 to December 2020 were selected. This study complied with the relevant requirements of the Declaration of Helsinki of the World Medical Association. All patients signed an informed consent form and were approved by the Ethics Committee of Huangshi Central Hospital (approval No. 2020–18).

### Selection criteria

Inclusion criteria included the following: (1) patients who met the diagnostic criteria for stroke established by the Fourth Academic Conference on Cerebrovascular Disease (10) using cranial CT or MRI; (2) patients with stable disease (stable vital signs and no further symptoms) who could move appropriately at the bedside; patients whose standing balance reached grade II and was able to walk more than 10 m with assistance; (3) patients without serious heart, liver, kidney, and other organ diseases; and (4) informed consent and signed informed consent were gained.

Exclusion criteria: (1) patients with disorders of consciousness or communication skills; (2) patients who were unable to get out of bed due to other reasons for limb movement disorders; (3) patients with other neurological diseases, damaged vestibular, and cerebellar dysfunction; (4) patients with lower extremity with bone and joint disease; (5) patients who were bedridden for a long time; (6) patients with malignant tumors; (7) patients who participated in other forms of rehabilitation training that had an impact on the results of this study; and (8) patients who could not complete follow-up or were lost to follow-up.

### Nursing methods

#### High falls risk screening

Falls risk was predicted using the Morse Fall Scale for identifying fall risk factors for stroke (11). The scale has 6 dimensions: mental status (0–15 points), gait (0–20 points), intravenous therapy/heparin lock (0–20 points), ambulatory aids (0–30 points), secondary diagnosis (0–15 points), and history of falling (0–25 points). The total score is 0–125 points; the higher the score, the higher the risk of falling on the affected limb.

#### Conventional recovery methods

(1) All patients were given health education for fall prevention and related facilities (including crutches, bathroom handrails, and non-slip shoes); patients were encouraged to get out of bed as soon as possible, and patients and their families were instructed on how to use wheelchairs and beds correctly. Additionally, patients were urged to use non-slip shoes to get out of bed and walk. (2) With limb positions in supine and sitting positions, continuous passive stretching of limbs for antispasmodic treatment was given; all joints of the affected limb should be passively moved, and the range of motion should not cause pain to the patient. (3) Sensory stimulation was given, including stretching the muscles briskly, tapping and brushing the tendon belly, and squeezing the muscle belly; the joint response and joint movement of the upper and lower limbs were used to induce the movement of the affected limb. (4) Patients received both double-bridge practice and single-bridge practice. (5) Shoulder exercises: passive and active activities of the shoulder joint, induction and control of the movement of the shoulder joint in all directions, mainly in front of the shoulder, shoulder abduction, and shoulder external rotation. (6) Upper limb training: the induction and control of elbow joint movement, mainly the extension of the elbow and the supination of the forearm. (7) The movements of the wrist and finger joints mainly included the dorsal extension of the wrist, lateral deviation around the ulna, thumb abduction, and pairing of the fingers. (8) Lower extremity exercises: induction and control of hip and knee joint movements in all directions in supine and prone positions, prevention of foot drop and varus, and maintenance and control of limb position.

### The control group received basic nursing combined with activity aids intervention

The auxiliary device was composed of a toilet plate, hinge, support rod, table plate, and fixing belt. As a set of combined devices, it is suitable for all high-risk and semi-disabled people, as well as patients with various potential risk factors for falls. By using the auxiliary device, the occurrence of falling events could be reduced or avoided, and it was convenient to use, detachable, and easy to assemble.

**Figure 1:**
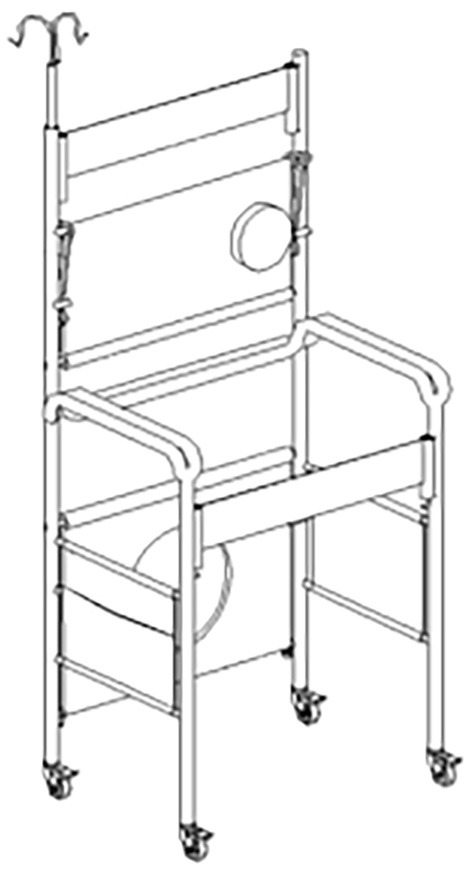
Schematic diagram of the auxiliary device.

The assistive devices were available for combined usage freely according to the different needs of patients. According to different activity needs, the upper table can be used by the bedside to help with eating and placing items and the lower toilet plate can be used as a cushion. After recovering the table and toilet plate, the patient can move out of bed and walk with the help of the device. Installing a stretch muscle strength exercise elastic belt in the seat frame can help patients do resistance exercises to enhance the muscle strength of the upper limbs. When infusion occurs, the hook can be opened to suspend the liquid. When going out, the water cup, obligation, and drainage bag can be put on both sides after hanging the cloth bag on the device. Therefore, it has strong practicability and can also be applied to the intervention period. The device was used for 5–6 h a day for 2 months (Figure 1).

### The study group was given a balance training intervention

The patients were given gait balance training in addition to the intervention of the control group, which was demonstrated by the uniformly trained nursing staff, and the movements of the patients were guided and corrected one-to-one to avoid falls accidentally during the exercise to ensure the safety of the patients. Preparation before exercise: the venue was spacious and quiet, with appropriate temperature and humidity; the floor was dry and non-slip, and the patients wore loose clothes and non-slip shoes. The specific training methods of balance training included a total of seven types of movements, including standing with eyes open, standing with eyes closed, center of gravity transfer, squatting with knees bent, body rotation, side start, and standing heel raises. The balance ability and the coordination of the main muscle groups of the lower limbs of patients were exercised. Among them, the turning action and the side start were divided into two directions, left and right, and the center of gravity transfer was divided into four actions: front, back, left, and side. Each exercise was carried out two times for a total of 15 min, and the training was carried out seven times a week for 2 months.

### Observation indicators

Balance function was assessed using the Brunel Rating Scale (BBA) (12) to evaluate before and after treatment. Lower extremity motor function was assessed using the simplified lower extremity Fugl-Meyer evaluation scale (13). Falls risk was assessed using the Morse Fall scale (14). We also monitored walking ability using the functional walking scale (FAC) (15) and the Timed Up and Go (TUG) test (16). All parameters were compared between before and 3 months after treatment. The hospitalization time and the number of falls after discharge for the two groups were recorded. For quality control, scores were counted by the same two-blinded qualified therapists with 8 years of experience.

### Statistical methods

The SPSS 21.0 software was used to analyze the data. Scores such as balance function, lower extremity motor function, falls risk, functional walking ability, and hospitalization time were expressed as x ± s, with the use of a *t*-test. The counting data such as the number of falls were expressed as a rate (%), and the chi-square (χ^2^) test was applied. *P* < 0.05 was considered to be statistically significant.

## Results

### General information

A total of 112 patients were included in the two groups, with 56 cases in each group, including 65 men and 47 women. There was no statistical difference in general information such as age, gender, course of the disease, stroke type, and side between the two groups (*P* > 0.05) (Table 1).

**Table 1:** Comparison of general data between the two groups.

**Index**		**Experimental group (*n* = 56)**	**Control group (*n* = 56)**	**χ2/t**	**P**
F/M (*n*)		32/24	33/23	0.037	0.848
Age (year)		57.87 ± 15.02	57.78 ± 14.97	0.032	0.975
Course (d)		42.87 ± 10.27	42.81 ± 10.23	0.031	0.975
Stroke types	Intracerebral hemorrhage	17 (30.36%)	15 (26.79%)	0.175	0.676
	Cerebral infarction	39 (69.64%)	41 (73.21%)		
Stroke side	Left	42 (75.00%)	40 (71.43%)	0.182	0.669
	Right	14 (25.00%)	16 (28.57%)		
Station balance level	II	36 (64.29%)	34 (60.71%)	0.152	0.696
	III	20 (35.71%)	22 (39.29%)		
Number of falls (times) in the past 6 months		0.98 ± 0.12	1.02 ± 0.13	−1.692	0.093

### Balance function scores

**Figure 2:**
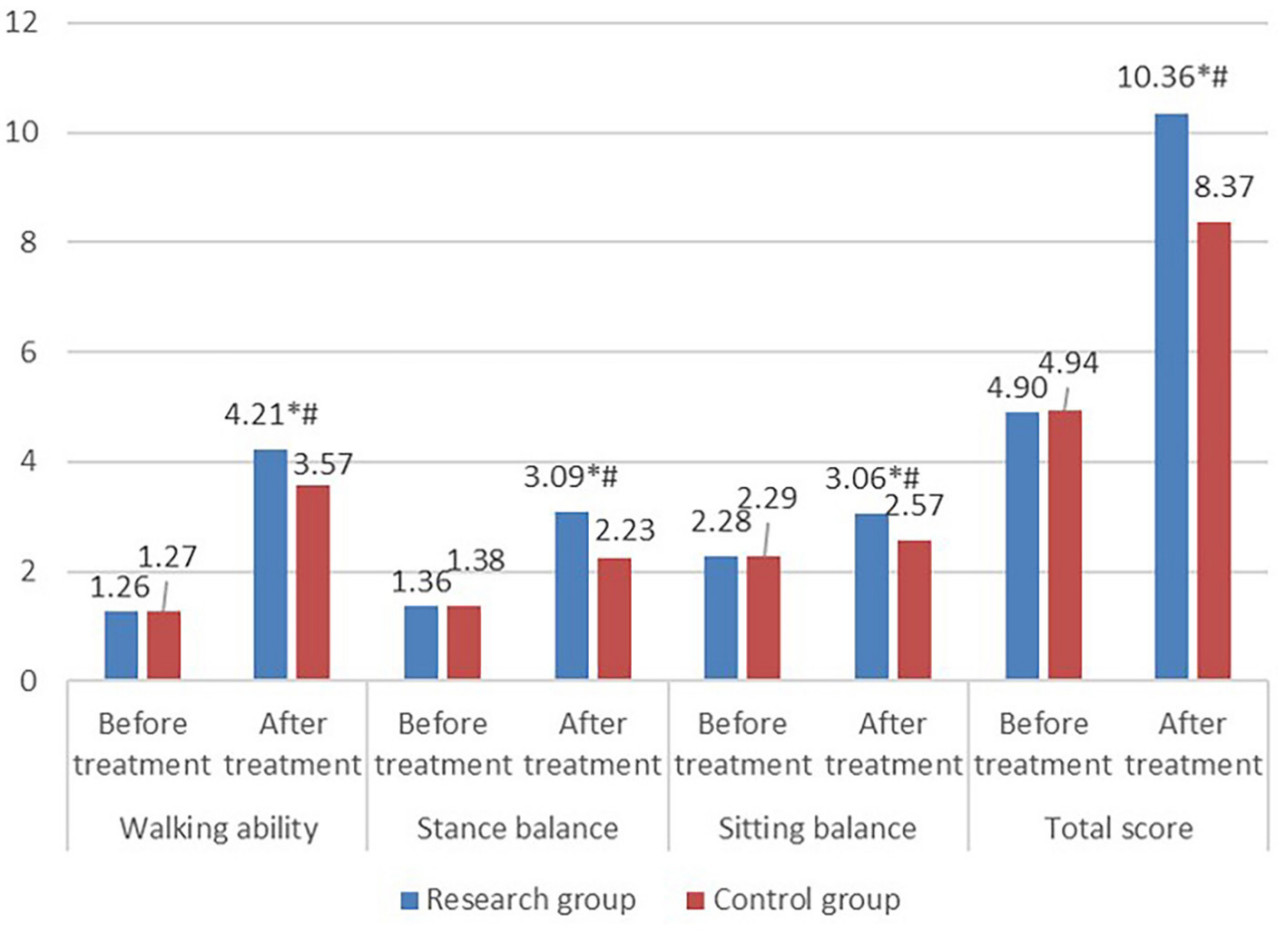
Comparison of balance function scores between the two groups. Balance function in the research group including walking ability, stance balance, sitting balance, and total score improved after treatment. Additionally, balance function of the research group after treatment was significantly better than that of the control group.

Before treatment, there was no significant difference in balance function scores between the two groups (*P* > 0.05). However, after treatment, a higher balance function score of the study group was seen than that of the control group, and the difference was statistically significant (*P* < 0.05) (Figure 2).

### Lower extremity motor function scores

**Figure 3:**
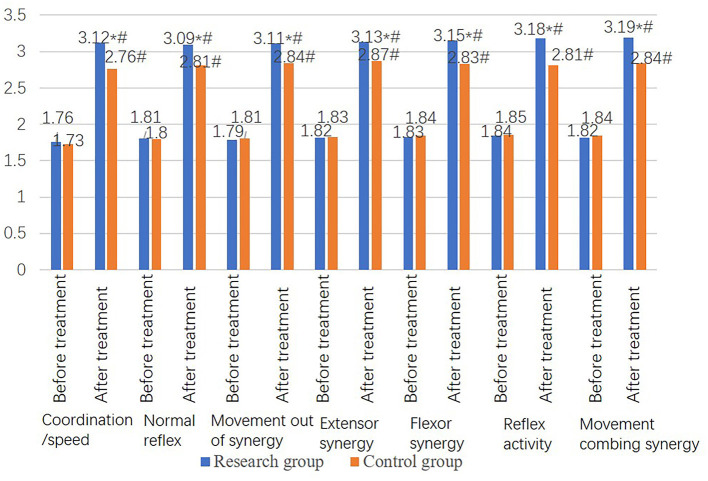
Comparison of lower extremity motor function scores between the two groups. Lower extremity function in the research group including coordinate/speed, normal reflex, movement out of synergy, extensor synergy, flexor synergy, reflex activity, and movement combining synergy improved after treatment. Additionally, lower extremity motor function of the research group after treatment was significantly better than that of the control group.

Before treatment, no significant difference was found in lower extremity motor function scores between the two groups (*P* > 0.05). However, the lower extremity motor function score in the study group was significantly higher than that in the control group after treatment (*P* < 0.05) (Figure 3).

### Fall risk scores

**Figure 4:**
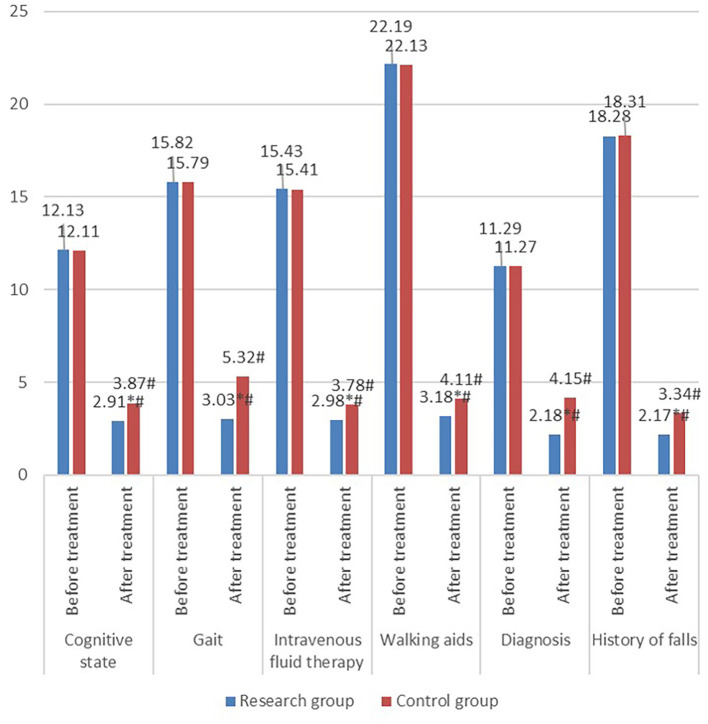
Comparison of fall risk scores between the two groups. Fall risks in the research group including cognitive state, gait, intravenous therapy fluid, walking aids, diagnosis, and history of falls improved after treatment. Additionally, fall risks of the research group after treatment was significantly better than that of the control group.

Fall risk scores failed to show a significant difference between the two groups before treatment (*P* > 0.05), whereas it was evidently lower in the study group than that in the control group after treatment (*P* < 0.05) (Figure 4).

### Walking ability

**Figure 5:**
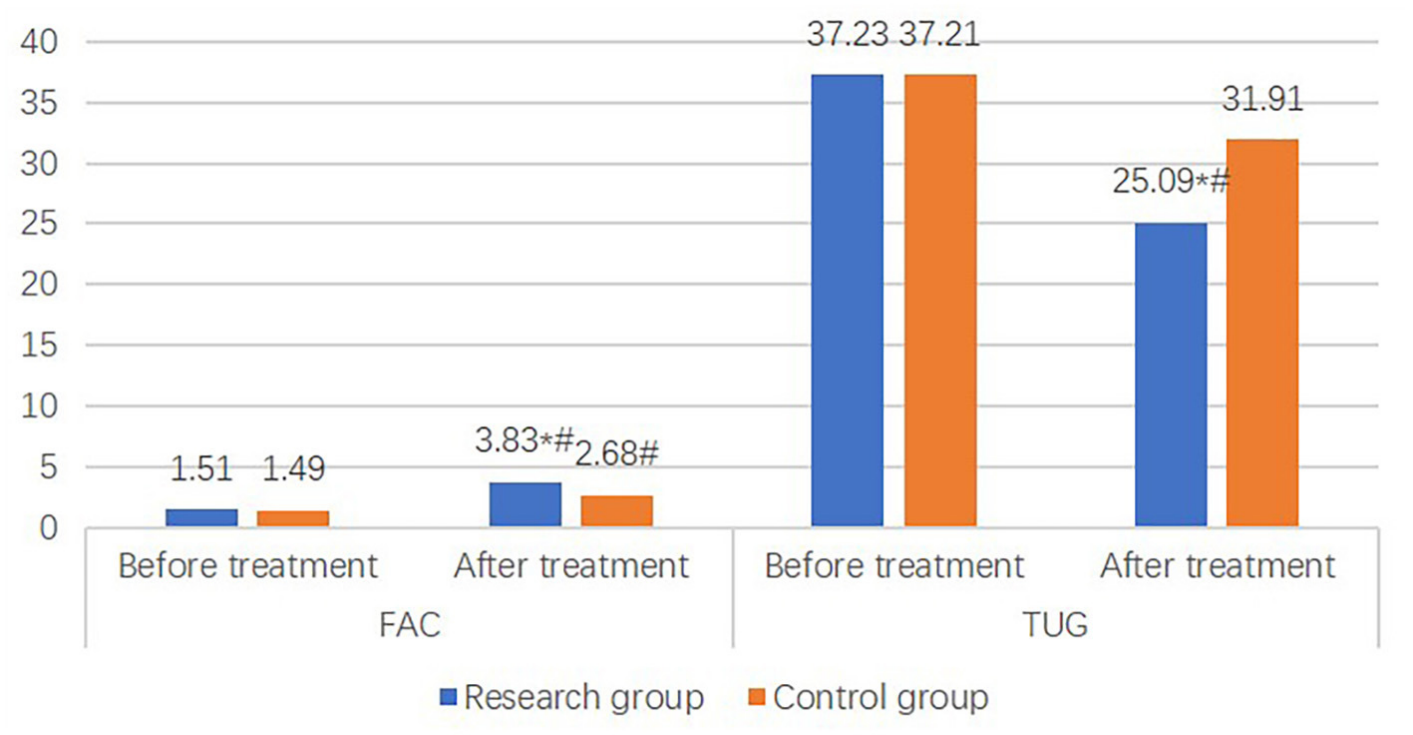
Comparison of walking ability between the two groups. Walking ability in the research group including functional walking scale, timed Up & Go improved after treatment. Additionally, walking ability of the research group after treatment was significantly better than that of the control group.

Before treatment, no significant difference was shown in FAC and TUG between the two groups (*P* > 0.05). However, after treatment, the FAC and TUG of the study group were significantly higher than those of the control group (*P* < 0.05) (Figure 5).

### Hospitalization time and frequency of falls

The hospitalization time and the frequency of falls in the 3-month follow-up in the study group were evidently lower than those in the control group (*P* < 0.05). But no significant difference was found in the frequency of falls between the two groups during hospitalization (*P* > 0.05) (Table 2).

**Table 2:** Comparison of hospital stay and frequency of falls between two groups.

**Groups**	***N***	**Hospitalization time (d)**	**Frequency of falls (times)**	
			**During hospitalization**	**During follow-up**
Experimental group	56	9.87 ± 2.01*^#^*	0	1*^#^*
Control group	56	12.89 ± 2.06	2	8

Compared with the control group after treatment (^#^P < 0.05).

## Discussion

Falls have been acknowledged as the most common complication and adverse event among patients with stroke. Patients with post-stroke exhibit impaired gait and balance function, and asymmetric gait is prone to causing the affected limb to be at high risk for falls (17). According to previous studies, falls are the leading cause of hospital readmission and can occur at all stages after stroke, including the acute, chronic, and convalescent stages (18). Earlier research (19) shows that the economic and social burden caused by falls in patients with stroke in China is twice that of other countries (20). Therefore, patients harboring stroke at high risk for falls should seek early rehabilitation intervention in order to avoid a series of complications caused by falls and to improve their quality of life.

Currently, there are several tools and devices to help patients get out of bed, such as medical out-of-bed aids. The auxiliary device is fixed on the adjacent, right-angled side of the hospital bed. Although it can safely help the patient get out of bed, it cannot be used to move around (7). The devices for auxiliary activities in this study are connectable and combinable, which can be freely combined and used according to the different needs of activities. It has strong practicability and can also be applied during treatment. Jing et al. (21) demonstrated that the application of a walker combined with comprehensive rehabilitation intervention in elderly patients with lower extremity fractures had a significant effect, which could effectively improve knee joint function and promote the recovery of walking ability. Balance ability is closely related to skeletal muscle strength, skeletal muscle tension, and postural reflex activities, especially in the core muscle group of the human body (13). The balance exercise in this study consisted of seven movements, including standing with eyes open, standing with eyes closed, center of gravity transfer, squatting with knees bent, body rotation, side starts, and standing heel raises. Our study focused on exercising core muscles, including the lower back muscles, abdominal muscles, hip muscles, and lower extremity muscles (22). Based on the study supported by Wang Jie (22), balance training can effectively restore the balance function of stroke patients with hemiplegia. Consistent with the results of previous studies, this study showed that after treatment, the balance function score of the study group was significantly higher than that of the control group, suggesting that balance training in addition to auxiliary activity can effectively restore the balance function of patients with stroke at high risk for falls.

The simplified Fugl-Meyer assessment scale for the lower extremity is detailed and quantified for each part, which improves the reliability and validity of the assessment of limb motor function and facilitates clinical research and academic exchanges for students (23). In addition, considering the assessment of motor function for the lower extremity is closely related to balance and joint range of motion and reflects the interaction of various factors in the process of motor function recovery in patients with stroke, it is considered an effective assessment method (24). The study by Zhu Yan (25) found that balance function training combined with early rehabilitation nursing could effectively improve the balance function of stroke patients with lower limb hemiplegia and thereby concluded its beneficial outcome in the balance and gait of patients with hemiplegia after stroke. Consistent with previous studies, our results showed a significantly higher lower extremity motor function score for the study group than that of the control group after treatment, indicating that balance training in addition to activity aids was able to effectively improve the lower extremity motor function of patients with stroke at high risk for falls.

Patients with stroke are often accompanied by neurological damage symptoms. In clinical practice, most of the patients with stroke are over 40 years old, with some patients being accompanied by various cardiovascular diseases, which further increases the risk of falls. In addition, some patients with stroke receive a sedative, hypoglycemic, antihypertensive, and other drugs for a long time, with reduced response and cognitive function and an increased risk of falling (26). Therefore, the possibility of falls should be focused and monitored in patients with high-risk strokes. The study by Liu Wenwei (27) depicted that balanced cognitive training was able to help correct the abnormal gait of patients with stroke, promote the recovery of their independent walking function and activities of daily living, and reduce the incidence of falls. According to the research by Wang Xiaowei (28), balance function training had a positive impact on the prevention of falls in patients with stroke. Similarly, the results of this study found that after treatment, the falls risk score of the study group was significantly lower than that of the control group, suggesting that balance training in addition to auxiliary activity was able to markedly reduce falls risk in patients with stroke at high risk of falling.

Walking ability is an important ability of daily activities. It consists of walking ability on level ground and an auxiliary road. Good walking ability is highly associated with the independent living ability and quality of life of patients with stroke (29). FAC is a commonly used scale for evaluating functional walking, with high reliability and validity (14). TUG is applied as a screening and evaluation tool. During the test, the patient undergoes a series of functional activities such as standing up, walking, turning around, walking again, and then sitting down. The above functional activities are the most basic activity skills in the patient's daily life. To complete the TUG, the patient needs to go through activities relating to body control and dynamic balance, such as the stand-up response phase, the walking acceleration phase and deceleration phase, and turning around. The above process actually fully reflects the comprehensive ability of the patient's balance and movement (15). Therefore, TUG can be used as an evaluative test to reflect the dynamic balance and walking ability of patients with stroke. The study by Yeqing et al. (30) pointed to the use of suspension training to intervene with the core muscle group in patients with hemiplegia after stroke as beneficial to the improvement of the balance ability and walking ability of patients, which is worthy of clinical promotion (15). In total, these results are compatible with our findings that, after treatment, the FAC and TUG in the study group were significantly higher than those in the control group, revealing that balance training in addition to auxiliary activity had the potential to effectively improve the walking ability of patients with stroke at high risk for falls. This study analyzed the comparison of hospitalization time and the frequency of falls in patients at high risk for falls between the two groups. The results showed that the hospitalization time and the frequency of falls during the 3-month follow-up in the study group were significantly lower than those in the control group, suggesting that balance training in addition to auxiliary activity can shorten the hospitalization time and prevent falls in patients with stroke at high risk for falls. However, one patient fell within 3 months after discharge, mainly because the patient had been in bed for too long after returning home, and after he got out of bed and walked with a changed body position, the occurrence of dizziness, unsteady walking, and general weakness may have led to the fall.

The present study has the following limitations: First, given the small sample size included in a single-center study, further multi-center randomized controlled research with a larger sample size is warranted to confirm the current findings. Second, this study is limited to evaluating the clinical practice process with a short follow-up; more studies focusing on the best practice evidence on the incidence of falls in patients after discharge should be carried out.

Taken together, balance training in addition to auxiliary activity elicited beneficial outcomes in terms of effectively improving the balance function and lower extremity motor function of patients with stroke at high risk of falling. It also reduced the risk of falling and restored walking ability, which may have the potential for wide clinical application.

## Data availability statement

The original contributions presented in the study are included in the article/supplementary material, further inquiries can be directed to the corresponding authors.

## Ethics statement

The studies involving human participants were reviewed and approved by Ethics Committee of Huangshi Central Hospital. The patients/participants provided their written informed consent to participate in this study.

## Author contributions

HT, ZG, and SX were involved in conceiving the idea, study design, data analysis, interpretation, and writing up the manuscript. LC and HL were involved in the study design, data analysis, and writing of the manuscript. LX contributed to the study design, data analysis, revision of the manuscript, and responsible for the overall content as the guarantor. The final manuscript was read and approved by all authors.
